# Supplementary material for: Mutations in PCYT2 disrupt etherlipid biosynthesis and cause a complex hereditary spastic paraplegia
Source: Brain. 2019 Oct 22;142(11):3382–97. doi: 10.1093/brain/awz291 (PMC6821184; doi:10.1093/brain/awz291)
Supplement: awz291_Supplementary_Data [file awz291_supplementary_data.zip › awz291-Suppl_data/Supplementary_Data4.zip!/Supplementary information 4 - plasma lipidomics.zip!/Plasma lipidomics/Control vs Patient3a/Statistical plots and data/DG[O] species/Boxplots.pdf]

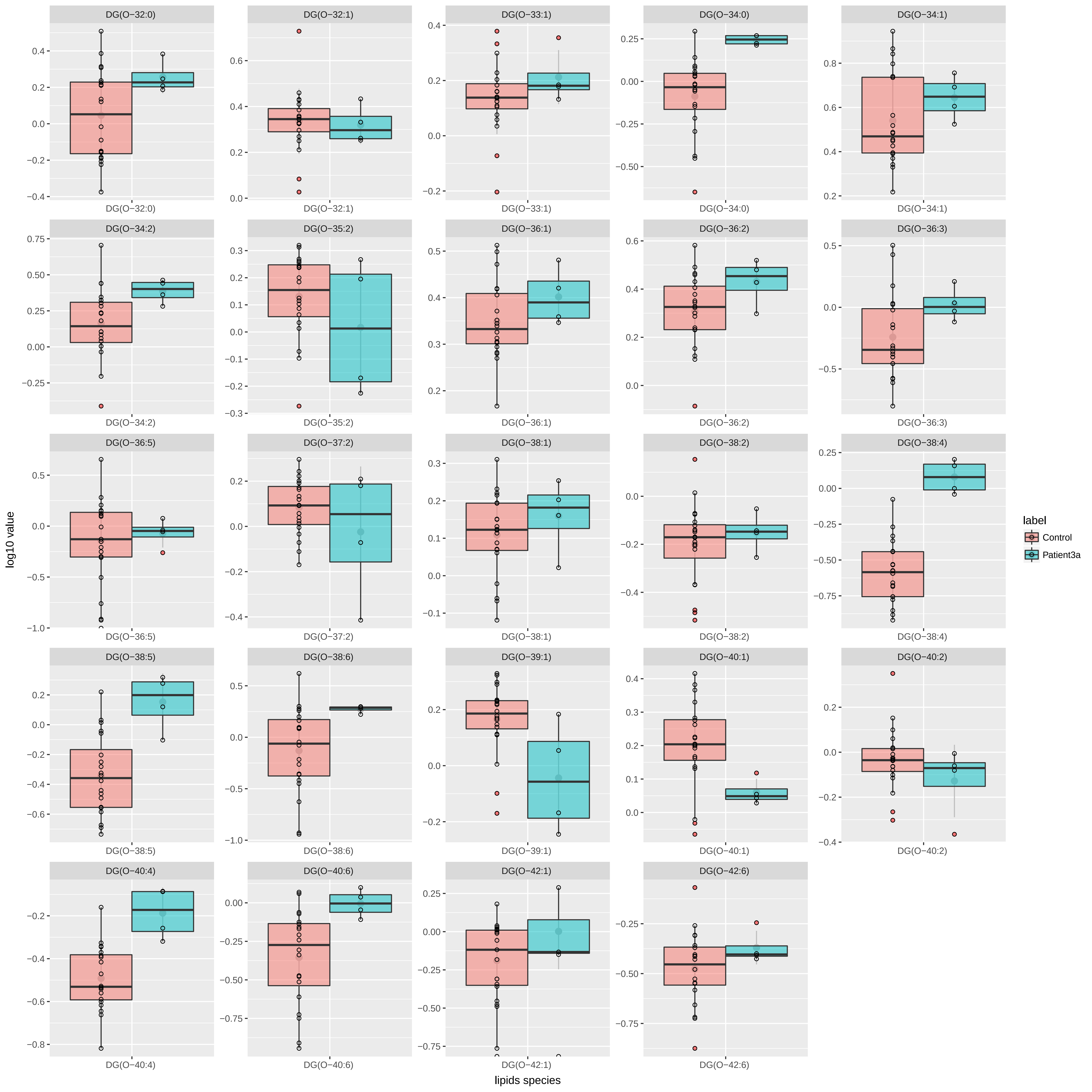

DG(O−32:0)
DG(O−32:1)
DG(O−33:1)
DG(O−34:0)
DG(O−34:1)
DG(O−34:2)
DG(O−35:2)
DG(O−36:1)
DG(O−36:2)
DG(O−36:3)
DG(O−36:5)
DG(O−37:2)
DG(O−38:1)
DG(O−38:2)
DG(O−38:4)
DG(O−38:5)
DG(O−38:6)
DG(O−39:1)
DG(O−40:1)
DG(O−40:2)
DG(O−40:4)
DG(O−40:6)
DG(O−42:1)
DG(O−42:6)
log10 value
lipids species
label
Control
Patient3a
